# Retrotranspositions in orthologous regions of closely related grass species

**DOI:** 10.1186/1471-2148-6-62

**Published:** 2006-08-16

**Authors:** Chunguang Du, Zuzana Swigoňová, Joachim Messing

**Affiliations:** 1 Waksman Institute of Microbiology, Rutgers University, Piscataway, NJ 08854, USA; 2 Department of Biology & Molecular Biology, Montclair State University, Montclair, NJ 07043, USA; 3 Department of Medical Genetics, University of Pittsburgh, Pittsburgh, PA 15213, USA

## Abstract

**Background:**

Retrotransposons are commonly occurring eukaryotic transposable elements (TEs). Among these, long terminal repeat (LTR) retrotransposons are the most abundant TEs and can comprise 50–90% of the genome in higher plants. By comparing the orthologous chromosomal regions of closely related species, the effects of TEs on the evolution of plant genomes can be studied in detail.

**Results:**

Here, we compared the composition and organization of TEs within five orthologous chromosomal regions among three grass species: maize, sorghum, and rice. We identified a total of 132 full or fragmented LTR retrotransposons in these regions. As a percentage of the total cumulative sequence in each species, LTR retrotransposons occupy 45.1% of the maize, 21.1% of the rice, and 3.7% of the sorghum regions. The most common elements in the maize retrotransposon-rich regions are the copia-like retrotransposons with 39% and the gypsy-like retrotransposons with 37%. Using the contiguous sequence of the orthologous regions, we detected 108 retrotransposons with intact target duplication sites and both LTR termini. Here, we show that 74% of these elements inserted into their host genome less than 1 million years ago and that many retroelements expanded in size by the insertion of other sequences. These inserts were predominantly other retroelements, however, several of them were also fragmented genes. Unforeseen was the finding of intact genes embedded within LTR retrotransposons.

**Conclusion:**

Although the abundance of retroelements between maize and rice is consistent with their different genome sizes of 2,364 and 389 Mb respectively, the content of retrotransposons in sorghum (790 Mb) is surprisingly low. In all three species, retrotransposition is a very recent activity relative to their speciation. While it was known that genes re-insert into non-orthologous positions of plant genomes, they appear to re-insert also within retrotransposons, potentially providing an important role for retrotransposons in the evolution of gene function.

## Background

Retrotransposons replicate intracellularly through reverse transcription of their RNA and integration of the resulting cDNA into another locus of the host genome. Two main groups of retrotransposons are recognized: the long terminal repeat (LTR) retrotransposons that have long terminal repeats at both ends; and the non-LTR retrotransposons that are lacking terminal repeats but contain a polyadenylate sequence at their 3' termini. LTR retrotransposons are the most abundant components of eukaryotic genomes. The two major classes are *Ty1-copia *and the *Ty3-gypsy *elements. Both *Ty1-copia *and *Ty3-gypsy *elements contain two major genes, *gag *and *pol*. These genes produce polyproteins, which are subsequently cleaved into functional peptides by an element-encoded protease. The *gag *gene encodes structural proteins important for the packaging of retrotransposonal RNA, while the *pol *gene encodes enzymes essential for the retrotransposon life cycle [1].

While mammalian genomes largely have the non-LTR retrotransposons, such as Alu repeats with more than 1 million copies comprising roughly 10% of the human genome [2], plants contain mainly LTR retrotransposons. Moreover, it appears that while mammalian genomes vary within a narrow range in their genome sizes, plant genomes vary extensively in part due to the differential amplification of LTR retrotransposons in different species. For instance, rice is about six times smaller than maize and its content of class I elements, excluding non-LTR retrotransposons, is about 18% while in the maize genome it represents 55% [3]. It also has been suggested that genome size can decrease due to deletion of class I elements [4]. The ubiquity of LTR retrotransposons in plant genomes is also illustrated by the nesting effect [5], where a young element inserts into an older element, originally described for the *adh1 *locus in maize [6].

Mobile elements have shaped both genes and entire genomes [7]. They usually insert into intergenic regions and are silenced to prevent additional rounds of amplification. Nevertheless, it would be simplistic to assume that they do not play a functional role. Contrary to the previously anticipated lack of functionality of TEs, it appears that they are indeed part of the transcriptome of different plant species [8], which correlates well with the fact that a subset of retroelements is hypomethylated [3]. It also has been shown that specific retrotransposon families are found in centromeric regions and possibly play a role in centromere function [9]. Furthermore, TEs have been reported to affect gene expression. Recently, Tos17, a copia-like retrotransposon, was found to become active in rice tissue, but silenced when plants were regenerated [10]. Because new insertion events in regenerated plants become heritable, they have been mapped to the genome and found to function in gene inactivation [11].

Accumulation of completely sequenced genomes provides an unprecedented opportunity to study the contribution of TEs to gene structure and gene function. In *Caenorhabditis elegans *the majority of LTR retrotransposons are located in or near genes [12]. Computational analyses of the sequenced human genome indicate that retrotransposon sequences are located in the coding regions of at least 4% of the genes [13], and in the promoter regions of at least 25% of the genes [14]. In the *Drosophila melanogaster *genome, 2% of the genes (approximately 300 genes) are spatially associated with an LTR retrotransposon sequence (*i.e*., an LTR retrotransposon sequence is in or within 1,000 bp from a gene) [15].

Genomic structure and gene expression can be affected by DNA rearrangements, such as deletions or translocations, caused by retrotransposition. Given such range of activities and rapid amplification it has been suggested to apply a different substitution rate of 1.3 × 10^-8 ^mutations per site per year for plant LTR retrotransposons when compared to plant genes [4]. Therefore, previous estimates of retrotransposon insertions will have to be re-evaluated [5,16,17]. Furthermore, in previous studies, the analysis of the retrotransposon content has been confined to single chromosomal regions consisting mostly of single BAC clones [6,18-20].

**Figure 1:**
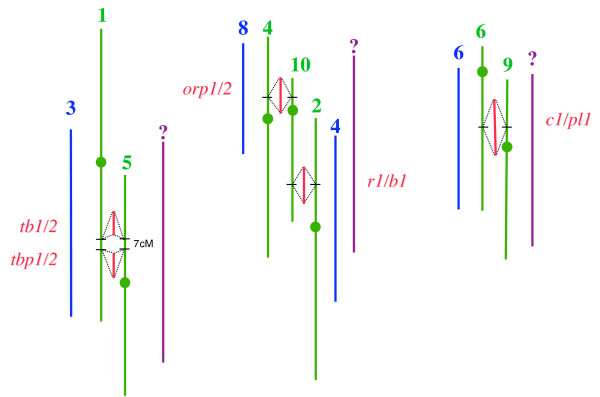
Five orthologous regions analyzed. Each maize region is represented by two homoeologous sequences depicting the whole-genome duplication event. Both rice and sorghum have one chromosomal sequence aligned. Each number on top of the line is the chromosome number. However, the chromosome number for sorghum is unknown. Blue, green, and purple lines represent rice, maize, and sorghum, respectively. A more detailed annotation of these regions with sequence coordinates have been published previously [31].

To better understand how transposable elements have influenced the evolution of chromosomal regions of common ancestry in plants we have examined the content of TEs and their times of insertion within five chromosomal intervals across three grass species: maize, sorghum, and rice (Fig. 1). Each region of tetraploid maize is represented by two homoeologous sequences depicting the whole-genome duplication (WGD) event. The orthology (common ancestry) of the studied regions was established from the structural alignment of orthologous genes [21]. Consistent with previous reports, we found that retrotransposons contribute to an increase in genome size in all three taxa; however, the intensity, spatiality, and directionality are considerably different among the grasses. An unexpected finding of intact genes within LTR retrotransposons highlights the possibility of their involvement in genomic rearrangements resulting in gene non-collinearity among related taxa.

## Results

### Comparisons of LTR retrotransposons in five orthologous regions of the maize, sorghum, and rice genomes

We selected a total of 30 genomic clones covering five chromosomal regions of common ancestry from the sorghum, rice, and maize genomes (Table 1). Each maize chromosomal region was represented by two homoeologous sequences as the products of WGD (Fig. 1). Within the five regions we identified a total of 132 LTR retrotransposons (Table 2). The LTR retrotransposons comprise 45.1%, 21.1%, and 3.7% of the genomic sequences of maize, rice, and sorghum, respectively. Unlike maize that contains LTR retrotransposons in all studied regions, the *orp1/orp2*, *r1/b1*, and *tbp1/tbp2 *regions of sorghum and *c1/p1*, *tb1/tb2 *regions of rice lack LTR retrotransposons. The number of LTR insertions per studied region is between 7 to 20 in maize, 0 to 6 in rice, and 0 to 2 in sorghum. In contrast to rice and sorghum a total of 7 solo LTRs have been identified in the maize regions. These solo LTRs are the remnants of full LTRs after recombination.

**Table 1:** Five genomic regions orthologous among maize, sorghum, and rice.

Region	Orthologs	Major Gene	Background	Chromosome location	Clone	Size (bp)	Accession
*orp1/orp2*	orp1	orange pericarp	Maize B73	4S	Z573F08	181627	AY555142
	Zmfiel1	Zmfie1 duplicate	Maize B73	4S	Z078P04	189000	AY560576
	orp2	orp1 duplicate	Maize B73	10S	Z573L14	144792	AY555143
	Zmfiel2	fie homolog	Maize B73	10S	Z273B07/Z409L08	138000	AY560578
	orph1	orp1 homolog	Sorghum Btx623	unknown	SB18C08	159669	AF466200
	fieh1	fie homolog	Sorghum Btx623	unknown	SB250O22	84604	AF466200
	rice ortholog		japonica	8	OJ1613-G04	136186	AP003896
	rice extension		japonica	8	P0680F05	17000	AP005620

*r1/b1*	r1	red color	Maize B73	10L	Z138B04	115734	AF466202
	r1 extension	3' extension	Maize B73	10L	Z333J11	207475	AF466202
	b1	booster	Maize B73	2S	Z092E12	147198	AF466203
	b1 extension	3' extension	Maize B73	2S	Z556K20	90000	AY542310
	rh1	r homolog	Sorghum Btx623	unknown	SB20O07	157237	AY542312
	rice ortholog		japonica	4	OSJNBa0065O17	167446	AL606682
	rice extension		japonica	4	OSJNBb0012E24	127506	AL606647

*c1/pl1*	c1	colored aleurone	Maize B73	9S	Z438D03	184890	AY530950
	c1 extension	3' extension	Maize B73	9S	Z214A02	159000	AY530951
	pl1	purple plant	Maize B73	6L	Z576C20	155173	AY530952
	pl1 extension	3' extension	Maize B73	6L	Z264N17	161000	AY560577
	ch1	c1 homolog	Sorghum Btx623	unknown	SB35P03	144120	AF466199
	rice ortholog		japonica	6	OSJNBb0015B15	123160	AP005652

*tb1/tb2*	tb1	teosinte branched	Maize B73	1L	Z178A11	130843	AF464738
	tb1 extension	5' extension	Maize B73	1L	Z013I05	152337	AY325816
	tb2	tb1 duplicate	Maize B73	5S	Z195D10	141937	AF466646
	tbh1	tb1 homolog	Sorghum Btx623	unknown	SB45119	77947	AF466204
	rice ortholog		japonica	3	OSJNBa0004G17	139071	AC091775

*tbp1/tbp2*	tbp1	TATA-binding protein	Maize B73	1L	Z477F24	212000	AY542798
	tbp2	tbp1 duplicate	Maize B73	5S	Z474J15	194000	AY542797
	tbph1	tbp1 homolog	Sorghum Btx623	unknown	SB32H17	100707	AF466201
	rice ortholog		japonica	3	OSJNBa0075A22	153828	AC133859

**Table 2:** Comparison of LTR retrotransposons in orthologous regions of maize, rice, and sorghum

Region	Marker Genes	Length (Kb)	LTR	Nested LTR		Fragemented	Solo LTR
				Single Layer	Multi-Layer	LTR	

*orp1/orp2*	orp1	358	20	yes	yes	1	1
	orp2	286	11	yes	yes	2	1
	sorghum ortholog	202	0	no	no		
	rice ortholog	133	6	no	no		

*r1/b1*	r1	290	18	yes	no		
	b1	206	12	yes	no		2
	sorghum ortholog	157	0	no	no		
	rice ortholog	250	2	no	no		

*c1/pl1*	c1	331	14	yes	yes	1	2
	pl1	316	11	yes	yes	2	
	sorghum ortholog	144	2	yes	no		
	rice ortholog	123	0	no	no		

*tb1/tb2*	tb1	220	11	yes	no	2	
	tb2	141	7	yes	no	1	1
	sorghum ortholog	78	1	no	no		
	rice ortholog	139	0	no	no		

*tbp1/tbp2*	tbp1	212	7	yes	yes		
	tbp2	194	9	yes	yes	2	
	sorghum ortholog	100	0	no	no		
	rice ortholog	153	1	no	no		

Total	maize	2554	120	yes	yes	11	7
	sorghum	681	3	yes	no	0	0
	rice	798	9	no	no	0	0
	all together	4033	132			11	7

The cumulative length of the five pairs of homoeologous regions in maize is 2,554 kb, within which we identified 120 LTR retrotransposons. Therefore, on average, there is one LTR retrotransposon every 21.3 kb of genomic sequence. The two major classes of those are copia-like LTR retrotransposons with 39.0% and gypsy-like LTR retrotransposons with 36.9% (Table 3). The most abundant LTR retrotransposon families in these regions are *Huck*, *Ji*, and *Opie *accounting for 24.8%, 21.5%, and 14.3%, of the LTR retrotransposons, respectively. The average size of intact *Huck*, *Ji*, and *Opie *retrotransposon is 14.7 kb, 9.4 kb, and 9.0 kb, respectively. These three LTR retrotransposons, occupying 698.7 kb, represent 27.4% of the 2,554 kb genomic sequence of maize. Similar estimates were obtained from randomly sheared DNA end-sequences [22]. Because the chromosomal regions analyzed in this study were selected according to their known genetic markers, one could expect lower retrotransposon density than in any other genomic region. On the contrary, it appears that retrotransposons are not clustered in any region of the maize genome, but have penetrated euchromatic and heterochromatic regions on a random basis.

**Table 3:** Distributions of LTR retrotransposons in five duplicated regions of maize.

Regions	*Copia*				*Gypsy*					Others	Total
	*Ji*	*Opie*	*Fourf*	*Hoscotch*	*Huck*	*Grande*	*Cinful*	*Tekay*	*Milt*		

*orp1*	4	5			3	1	3		1	3	20
*orp2*	5	1			2				2	1	11
*r1*	8	3	1		2			1		3	18
*b1*	3	2			5	1			1		12
*c1*	2	4			3		1	1		3	14
*pl1*	5	1	1		3					1	11
*tb1*	2			1	1	1	1		1	4	11
*tb2*	1				1		1			4	7
*tbp1*	2	1			3					1	7
*tbp2*	2	3			1					3	9

Sum	34	20	2	1	24	3	6	2	5	23	120

											
Total Length	425 kb (*copia*-like)				449.41 kb (*gypsy*-like)						
	39.01% (*copia*-like)				36.89% (*gypsy*-like)						

### Nested LTR retrotransposons

**Figure 2:**
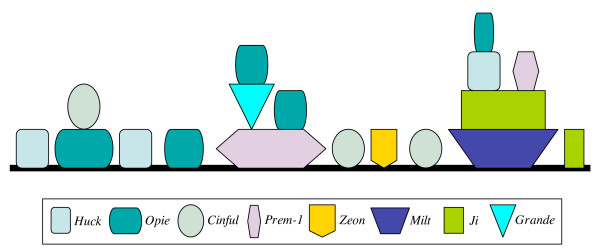
Organization of LTR-retrotransposons at the *orp1* locus. The maize *orp1 *region on maize chromosome 4S has three-layer nested LTR retrotransposons. This region also contains the highest number of LTR retrotransposons. A color code for the various retrotransposon families has been added.

The first genomic regions of the maize genome that have been sequenced contain both nested LTR retrotransposons as well as single retrotransposons [23,24]. The same pattern extends to the maize regions studied here. In two of the sorghum regions we identified three LTR retrotransposons, two of which were nested within the *c1/pl1 *region (Table 2). In rice only three of the five studied regions contained LTR retrotransposons and none of them appeared to be nested. This finding differs from earlier studies of other rice regions [25], indicating that within the rice genome there are islands of various intensities of transposition activity. Out of 132 LTR retrotransposons identified in the studied regions, 36 are nested within another LTR retrotransposon (Table 2). The maize *orp1 *region contains the largest number of LTR retrotransposons and also holds a three-layer nested LTR-retrotransposon structure (Fig. 2). Apparently, these nested structures are the primary source of gene density reduction in chromosomal regions. Although an LTR retrotransposon can insert into another member of the same family (*Ji *inserted into *Ji*), most insertions are heterogeneous (*Opie *into *Ji *or *Cinful *into *Opie*). Events causing chromosomal reduction, contemporaneous to chromosomal expansion, are deletions of LTR retrotransposons as exemplified by the *Ji *solo LTR nested in a *Prem-1 *element within the maize *tb2 *region of chromosome 5S.

### LTR-retrotransposon insertion times

Integration of an LTR retrotransposon requires duplication of the LTR sequence. Therefore, the two LTRs of an inserted element are identical at the time of insertion. As time passes, nucleotide substitutions cause sequence divergence between the two LTRs. If the substitution rate is known, then the date of insertion can be estimated from the amount of divergence between the two LTRs [16]. In a recent study of the rice genome, Ma and Bennetzen [4] showed that the average level of nucleotide substitution in intergenic regions (1.18%) is about 2-fold higher than that of synonymous substitution in coding regions of genes (0.58%). Therefore, we applied a substitution rate of 1.3 × 10^-8 ^mutations per site per year as described previously [26] and calculated the time of insertion of the LTR retrotransposons identified within the studied segments [see Additional file 1]. Although among nested retrotransposons the insertion time of an internal LTR retrotransposon should be younger than the recipient one, there appeared to be a few exceptions in the maize regions. However, when we calculated 95% confidence intervals for the time estimates of LTR insertions using the MEGA2 program [27], the estimates for the LTR retrotransposons that are embedded in other LTR retrotransposons exhibit overlapping intervals with the recipient elements [see Additional file 1]. Therefore, we cannot be certain whether there are exceptions to the general pattern of a younger LTR retrotransposon inserting to an older LTR retrotransposon.

The *orp1/2 *regions contain most of the older LTR retrotransposons. Among these are the *Ji-1 *from the maize *orp2 *region (~2.5 mya), the *Dagul *(~3.8 mya) from rice, and also the oldest LTR retrotransposon within the studied segments, the *Prem-1 *from maize *orp1 *region (~4.6 mya). The *Ji-5 *and *Fasu *from the *r1 *region, the *Prem-1 *and *Ji *in the *c1 *region, and the *Yemi *in the *tb1 *region (all from maize) are the most recently inserted LTR retrotransposons. All LTR retrotransposons identified within the *c1/p1 *and *tbp1/2 *regions inserted less than 2 mya. All the LTR retrotransposons identified in the rice and sorghum fragments inserted within the last one million years. About 75% of the 108 intact LTRs inserted into their host genome less than 1 mya.

### Contribution of LTR retrotransposons to gene order in the maize genome

In most cases, we found that retrotransposons were interspersed between genic sequences; however in 2 of the 10 maize regions (the *orp2 *and *tbp1*) we detected a total of 11 predicted genes or gene fragments that were embedded within LTR retrotransposons. These accounted for 15% of all putative genes in these regions. To further investigate the nature of these insertions, we looked at these two regions in more detail.

**Figure 3:**
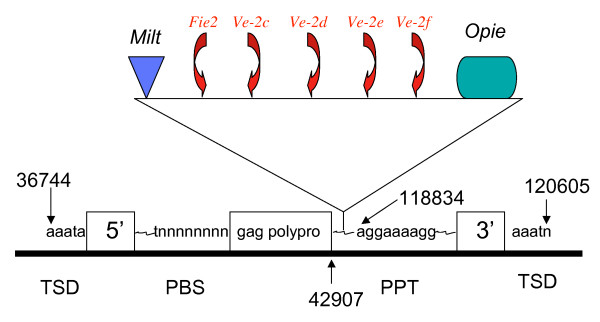
Genes nested in LTR retrotransposons at the *orp2 *locus on maize chromosome 10S. Some of the screened contigs permitted the analysis of more complex retrotransposon blocks like the region around *orange pericarp 2 *(*orp2*) locus on maize chromosome 10S. This region contains nested as well as non-nested LTR retrotransposons. Five genes are nested within *Ji-3 *retrotransposon of the *orp2 *region. Arrows provide the polarity of genes. Genes and elements are directly labeled in the figure. Position of insertions and truncations are given in nucleotide positions as they relate to the entire BAC sequence.

First we analyzed the *orp2 *region where we found one intact and four fragmented genes nested in a *Ji-3 *retrotransposon (Fig. 3). To reconstruct the history of insertion events, the nucleotide sequence of this *Ji-3 *retrotransposon was aligned with the full-length consensus sequence of a *Ji *element that was obtained from multiple sequence alignments of all *Ji *elements identified in the studied regions with additional sequences identified from database searches (see Methods). Based on this alignment, the *Ji-3 *retrotransposon has a length of 7,834 nucleotides after exclusion of inserted sequences between the target duplication sites (TDS) and arrived at this location about 2.5 mya. The polyprotein-coding region is truncated at position 42,907 (Fig. 3), where a complex set of sequences of different origins is inserted. This set contains a *Milt *element (at position 47,971) that arrived in its location about 0.385 mya, an *Opie *retrotransposon (ending at position 114,559) that inserted 0.308 mya, and, more importantly, it also includes five non-TE related genes of which one is intact. With these multiple insertions the *Ji-3 *element expanded by a total of 66,588 nucleotides. While insertion of retrotransposons into other retrotransposons is well documented from other regions of maize as well as other plant genomes, finding an intact gene embedded in a retroelement was unexpected. In contrary, the only example of the insertion of non-TE related genes into a retrotransposon are gene fragments embedded in a *huck *retrotransposon within the 9002 locus of the maize inbred line Mo17 that are unlikely to be functional [28].

Here, the intact gene found within the *Ji-3 *element belongs to a group of genes known to be important for seed development, also called the *Fie2 *(fertilization-independent endosperm-like) gene [29]. The four fragmented genes embedded within the *Ji-3 *retrotransposon are encoded by the opposite DNA strand compared to the *Fie2 *gene and are copies of the *Ve-2 *gene (verticillium wilt resistance-like) (*Ve-2c*, *d*, *e*, *f*) belonging to a group of disease resistance genes [30]. Interestingly, two intact copies of the *Ve-2 *gene reside in the upstream region outside of the *Ji-3 *element. Sequence comparison of the *Ve-2 *gene homologs from the rice genome uncovered a pattern of sequence fragmentation in the maize *Ve-2 *genes, indicative of independent truncations of all *Ve-2 *maize genes during or after their amplification. However, the lack of sufficient overlapping sequence homologies prevented us from performing phylogenetic analyses. The external position of two of the *Ve-2 *genes (*Ve-2a*, *b*) leads to the hypothesis that the *Ve-2 *genes internal to the *Ji-3 *element might have arisen from one or both of the *Ve-2 *genes external to the *Ji-3 *element. Perhaps, insertion of gene fragments in non-collinear chromosomal positions might be a more common theme as previously suggested [28].

For the intact *Fie2 *gene phylogenetic analysis [21] and expression data [29] are already available. There are two *Fie *genes in the maize genome that reside in homoeologous regions on chromosome 4 and 10 [31]. The orthologous regions in rice and sorghum each contain a tandem duplication of the *Fie *gene. Phylogenetic analysis of the *Fie *genes revealed that the two maize genes represent the two ancestral paralogs, indicating a deletion of two paralogous copies after the hybridization of the two maize progenitors [21]. Furthermore, the two tandem genes in rice and the *Fie1 *gene on maize chromosome 4 do not reside within a retrotransposon. Therefore, the *Fie2 *gene on the maize chromosome 10 must have been inserted from a close location in this genomic region into the *Ji-3 *element, but unlike the *Ve-2 *genes as intact gene. In addition, it is interesting to note the difference in expression of the two *Fie *genes in maize. The *Fie2 *gene, nested in an LTR retrotransposon, is expressed in the embryo sac before pollination, while the non-nested *Fie1 *gene on chromosome 4 is expressed exclusively in the endosperm of developing kernels at ~6 days after pollination [29]. It is unknown whether this difference in gene expression is based on the regulatory elements of the LTR retrotransposon flanking the *Fie2 *gene, but it has been suggested that LTR retrotransposons located in or near genes might alter gene expression and, therefore, contribute significantly to gene evolution [32].

**Figure 4:**
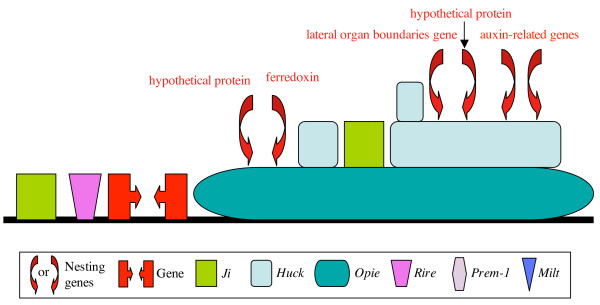
Genes nested in LTR retrotransposons at the *tbp1 *locus on maize chromosome 1L. The region around *TATA-binding protein 1 *(*tbp1*) on maize chromosome 1L contains nested as well as non-nested LTR retrotransposons. Four genes are nested within the *Huck *element and two within the *Opie *element of the *tbp1 *region. Genes are directly labeled in the figure. A color code for the TEs has been added.

The other region with a complex of predicted genes nested within LTR retrotransposons was the *tbp1 *region (Fig. 4). For further analysis, we selected the two copies of the intact auxin-related genes. To assess their abundance within the genome we searched the collection of maize GSSs, representing a high proportion of the maize genic regions, and found evidence for additional copies of these genes in other regions of the maize genome. Because these GSSs do not cover complete gene sequences, we only selected the two genes within the retroelement for comparison with homologs of the fully sequenced rice genome. Homologous genes are also found in many non-orthologous regions of the rice genome [31]. We estimated that the *Huck*-2 element arrived at this location (nested in an *Opie *element) about 0.2 mya, while the *Opie *element inserted in this region about 1.7 mya (Fig. 4). Phylogenetic analysis of the predicted auxin-related genes from this region with those identified in rice [see Additional file 2] revealed that the two maize genes were duplicated long before (> 50 mya) the insertion of the LTR retrotransposons, indicating that both genes existed in different positions of the maize genome and moved to this location recently.

## Discussion

### Contribution of LTR retrotransposons to plant genome evolution

Rice diverged from maize and sorghum about 50 mya [33] and sorghum diverged from maize about 12 mya [21]. After speciation, regions descending from ancestral chromosomes largely stayed intact for a long time as exemplified by retention of collinearity among these taxa [31]. Our results indicate that all three genomes independently experienced relatively recent LTR activity and their intensities varied among the different grass species. Compared to maize, rice has a relatively small genome with about 389 Mb. Although we did not find solo LTR retrotransposons in our studied rice regions, it has been reported that out of 1,219 LTR retrotransposons, 822 appeared to be fragmented elements [34]. The high rate of LTR retrotransposon deletions in rice may be one of the reasons that some rice regions may appear to have fewer nested LTR retrotransposons than the maize genome. Recently, it has also been suggested that the size of the rice genome decreased due to the higher proportion of solo LTRs versus intact retrotransposons [34]. The contraction coupled with the lack of expansion due to the lack of nesting could explain the differential retrotransposon density between maize and rice. Therefore, genome size differences in plants result from several mechanisms. If low density of retrotransposons in the orthologous regions of sorghum holds up on a genome-wide level, other mechanisms for the expansion of sorghum versus rice might still be uncovered. One interesting feature of retroelements is that they provide sites for additional insertions, which could have accelerated the expansion of the maize genome relative to rice.

### Association of LTR retrotransposons and functional genes

Given the abundance of retrotransposition events, the question arose as to how they might influence the expression of genes. Indeed, retrotransposons are involved in generating mutations through insertions near or within genes and affect their expression, usually in a negative fashion by decreasing or abolishing transcription of a gene or by detrimental alterations in transcript processing and/or stability. Retrotransposons inserted in or near plant genes have been reported in maize, rice, lettuce, wheat, tomato, tobacco, potato, and bell pepper [5]. Here, we have found that the predicted gene *RNAP II *in the maize *orp1 *region is only 415 bp away from the *Milt-1 *retrotransposon. Similarly to plant genomes retrotransposons seem to be important modulators of animal genomes as well. Approximately 2% of the genes (~300 genes) in the *Drosophila melanogaster *genome are associated with an LTR retrotransposon sequence [15]. In another case, the gene *Peg10 *is critical for mouse parthenogenetic development and provides the first direct evidence of an essential role of an evolutionarily conserved retrotransposon-derived gene in mammalian development [35].

### Gene movement within the maize genome

While numerous studies showed that LTR retrotransposons could arrive near or within genes, our finding of intact genes located inside LTR retrotransposons was unanticipated. Previous comparative studies already showed that non-collinear genes have moved to other locations within the same genome [31]. One of the possible mechanisms of gene movement appears to be based on the action of *helitrons *[36,37]. In such a case it is hypothesized that the replication of a sequence containing a gene is initiated by the action of a helicase similarly to the initiation of replication of single-stranded bacteriophage. This mode of replication is also referred to as rolling circle (RC) replication because the product is a single-stranded circular DNA. As a consequence one can expect that the donor site would stay intact and the extrachromosomal circular DNA gets integrated at another site by illegitimate recombination. However so far, *helitron *sequences appear to contain only gene fragments and not intact genes [37]. In case of the *Fie2 *gene, there is no donor site present in the rest of the genome. We have searched maize inbred lines for a haplotype progenitor that would contain a putative donor site for the *Fie2 *gene. However, when we compared about 40 different inbred lines, there was no line where the *Fie2 *gene site differed from inbred B73 (data not shown). Since a previous analysis of haplotype variability of the *z1C1 *locus suggested four major haplotypes among the core inbred lines, we would have expected that, if it exists, we would have found a second haplotype of the *fie2 *locus among the inbreds analyzed. Although we cannot be certain if the original site of the *Fie2 *gene was deleted after it moved into the retrotransposon or if the gene was excised and then re-inserted into the retrotransposon, its translocation mechanism appears to differ from the examples of *helitron*-based gene movements. Even with the retention of sequences at a donor site that contain genes, new gene copies do not have to arise from an RC-based amplification. For instance, gene insertions of copies of storage protein genes occurred during the last 5 million years in six different locations of the maize genome, relative to the rice genome by a mechanism that differs from *helitrons *[38]. Disease resistance genes are another example of recent insertions of gene copies into new chromosomal positions [39,40]. Whatever the mechanism for copying or excising genes might be, insertion into the genome requires a chromosome break. Perhaps retroelements are more prone to chromosome breakage, which would be consistent with the apparent layers of nested retroelements. Therefore, it would be conceivable that besides layered retroelements other sequences could insert within retroelements by illegitimate recombination.

Here we present a few interesting examples of intact genes inserted into an LTR retrotransposon, one of which is the *Fie2 *gene in the maize *orp2 *region (Fig. 2). Based on the conserved alignment of the tandem genes in the orthologous segments of sorghum and rice, and on the phylogenetic analysis showing that the two maize *Fie *genes represent the ancestral paralogs, we can conclude that the maize *Fie2 *gene itself or a copy of it must have translocated from its original orthologous position and ultimately arrived within the *Ji-3 *retrotransposon. Furthermore, the *Fie2 *gene is expressed in a specific pattern that differs from the *Fie1 *gene on the other homoeologous chromosomal region of maize. The duplication of the two *Fie *genes resulting from WGD in the progenitor of maize possibly led to four gene copies that were disadvantageous. It appears that a large percentage of duplicated genes in the maize genome lost their second copy [3]. The WGD possibly increased transposition and chromosomal breakages leading to the relocation of genes. The final positioning of the *Fie2 *gene within the LTR retrotransposon might have been advantageous in affecting the differential expression of the *Fie2*. Interesting is that the movement of the *Fie2 *gene has likely occurred over a very short distance on the same chromosome because it is located very close to its orthologous position.

Another possibility for the embedded genes is that processed gene transcripts of the embedded genes were co-packaged into the viral particles allowing the integration of the gene sequence into the body of the element by jumping templates via reverse transcription during the replicative process. This is less likely the case for short distance movement because integration of the composite element is likely to occur at an unlinked location. However, the second example described here, the auxin-related genes on chromosome 1 could have been derived from such a mechanism. If processed transcripts were co-packaged into viral particles, one would expect that those genes would be intronless. While the *Fie2 *gene on chromosome 10 contains its introns, the auxin-related genes on chromosome 1 do not have introns.

## Conclusion

In conclusion, we are providing here a new feature of how LTR retrotransposons are not merely parasitic in nature but have adapted to be elements in the genome that can rapidly rearrange the organization and possibly affect regulation of genes in response to the "challenge", as proposed by McClintock in 1984 [41]. As we show in this study, LTR retrotransposons contain intact gene copies that are much older than the time of the retroelement insertion within a genomic region. Such genes must have existed in another location of the genome prior to the LTR insertion. The sequences containing these genes were either copied or deleted from their original (orthologous) positions and then inserted into retroelements. Because retroelements do not excise from their position, but are copied and inserted into new genomic positions, they also could potentially place acquired gene copies throughout the genome, causing a disruption in gene order after speciation of ancestral chromosomes. If this were to be the case, one would expect that genes carried by retrotransposition would loose their introns from reverse transcription of processed transcripts as suggested for the auxin-related genes in *tbp1 *region of maize chromosome 1L. Therefore, it is possible that the frequent gene movements in grass genomes reported recently [31] could be explained in part by such a mechanism. This would resemble the proposed movement of gene fragments by mutator-related DNA transposable elements [42]. Furthermore, the short lifespan of LTR retrotransposons might explain why many sequences containing non-collinear genes might have lost the sequence motifs associated with retroelements from their flanking regions. In addition, nesting of genes in LTR retrotransposons might also result in the differentiation of the expression of duplicated genes. These findings support further studies to uncover the full extent of the effect of retrotransposons on the structural and functional evolution of genes and genomes.

## Methods

### BAC sequencing

We chose 18 maize BAC sequences from inbred line B73 and 6 sorghum BAC sequences from *Sorghum bicolor *cv. Btx623 [21]. We used maize genetic markers to identify orthologous clones from the *Oryza sativa *ssp. japonica cv. Nipponbare genome [31]. A data set comprising genomic sequences of five chromosomal segments from two homoeologous regions of the maize genome (resulting from WGD), one from sorghum and one from rice, have been previously aligned based on collinear genetic markers (Fig. 1). The data set consists of 2,554 kb of maize, 681 kb of sorghum, and 798 kb of rice compound sequences. The detailed information and accession numbers are listed in Table 1. The two previous reports have focused on the gene content of those regions. Here, we are using the same data set to examine the entire content of retrotransposable elements.

### LTR retrotransposon searches

LTR retrotransposons in plants are characterized by long terminal repeats (LTRs) that vary in size from a few hundred base pairs to several kilobases, and are generally terminated by the dinucleotides 5'-TG...CA-3' [5].

Step 1: Database mining – BAC sequences (Table 1) were searched against the nucleotide database of National Center for Biotechnology Information (NCBI), the repeat database that was recently established from all maize genomic sequences [3], and The Institute for Genomic Research (TIGR) repeat database, which also includes repetitive sequences from other plant species [43] using BLAST [44]. The cut-off values of e^-20 ^or less were used to select putative LTR retrotransposons. After comparing these three different blast results, we assembled a comprehensive list of LTR retrotransposons from all chromosomal regions.

Step 2: LTR searching – LTR_STRUC software was used to search the BAC sequences for full length and intact LTR retrotransposons [45]. The algorithm of LTR_STRUC program is based on important structural features, such as primary-binding site (PBS), polypurine tract (PPT), and the presence of canonical dinucleotides at the ends of each LTR (typically TG and CA).

Step 3: LTR matching – We extracted a collection of both 5' and 3' LTRs and used them as queries to search against original BAC sequences by using BLAST 2 Sequences [46]. We carefully checked the search results and sorted out all the possible LTR retrotransposons according to the estimated sizes of different types of LTR retrotransposons [22] for further sequence alignment. We also evaluated the structural characteristics of LTR retrotransposons, such as the presence of *gag *and *pol *genes.

Step 4: Sequence alignment – First we aligned all LTRs of the same class using ClustalX [47] and then performed phylogenetic clustering through maximum parsimony and maximum likelihood analyses using PAUP* 4.0b10 [48] to identify the probable paired LTRs. The paired LTRs were aligned again to recheck their accuracy. In addition, when pairs of LTRs were identified, internal structural characteristics such as PBS and PPT were examined.

### LTR retrotransposon insertion dates

Both LTRs of each identified LTR retrotransposon were aligned using ClustalX [47]. The distance estimations between pairs of LTR retrotransposons and their standard errors were based on the Kimura two-parameter (K2P) model as implemented in the MEGA-2 program [27]. Confidence intervals (CI) were calculated using the mean distance and the SE as estimated from K2P and thus they are symmetrical. In calculations of insertion times of LTR retrotransposons we used a mutational rate for intergenic regions of 1.3 × 10^-8 ^substitutions/site/year as described recently [26].

### Estimation of the time of gene duplication

For genes of interest, gene homologs from the rice and *Arabidopsis thaliana *genomes were identified by homology searches using BLAST [44]. Alignment of coding sequences performed by ClustalX [47] was visually reviewed. Phylogenetic analyses, including parsimony and maximum likelihood methods, were performed using PAUP* 4.0b10 [48]. To estimate the relative time of gene duplication we assumed that rice and maize diverged about 50 mya [49].

## Abbreviations

bacterial artificial chromosome (BAC), long terminal repeat (LTR), million years ago (mya), transposable element (TE), whole-genome duplication (WGD).

## Authors' contributions

CD and ZS performed the analysis of the data set. CD drafted the manuscript. ZS edited the manuscript. JM conducted the coordination of data analysis, the manuscript conception, and the writing of the manuscript. All authors read and approved the final manuscript.

## Supplementary Material

Additional file 1 Distance analysis of LTRs. The analysis provides the LTR retrotransposons, estimated distances (*k*) between pairs of LTRs, and times of insertion (mya). Click here for file

Additional file 2 Cladogram of auxin-related genes. The relationship of the auxin-related genes is presented as a cladogram resulting from maximum parsimony analysis using branch-and-bound search option. Click here for file
