# Supplementary material for: Retrotranspositions in orthologous regions of closely related grass species
Source: BMC Evol Biol. 2006 Aug 16;6:62. doi: 10.1186/1471-2148-6-62 (PMC1560396; doi:10.1186/1471-2148-6-62)
Supplement: Additional file 1 — Distance analysis of LTRs. The analysis provides the LTR retrotransposons, estimated distances (k) between pairs of LTRs, and times of insertion (mya). [file 1471-2148-6-62-S1.pdf]

Additional file 1 for "Retrotranspositions in orthologous regions of closely related grass species" by Du et al. BMC Evolutionary Biology #4916306119647011.

LTR retrotransposons, estimated distances (*k*) between pairs of LTRs, and times of insertion (mya).

orp1/2 **region**

LTRs	k (±SE)[a]	Time (95% C.I.)[b]
	maize chromosome 4S (*orp1*)	
Cinful-1	0.009 (±0.004)	0.346 (±0.300)
Cinful-2	0.023 (±0.006)	0.885 (±0.450)
Cinful-3	0.060 (±0.010)	2.308 (±0.750)
Huck-1	0.046 (±0.006)	1.769 (±0.450)
Huck-2	0.028 (±0.004)	1.077 (±0.300)
Huck-3	0.030 (±0.005)	1.154 (±0.375)
Ji-1	0.020 (±0.006)	0.769 (±0.450)
Ji-2	0.003 (±0.002)	0.115 (±0.150)
Ji-3	0.002 (±0.001)	0.077 (±0.075)
Ji-4	0.003 (±0.002)	0.115 (±0.150)
Opie-1	0.005 (±0.002)	0.192 (±0.150)
Opie-2	0.007 (±0.003)	0.269 (±0.225)
Opie-3	0.017 (±0.004)	0.654 (±0.300)
Grande	0.019 (±0.006)	0.731 (±0.450)
Milt	0.047 (±0.000)	1.808 (±0.000)
Prem-1-1	0.119 (±0.012)	4.577 (±0.900)
Prem-1-2	0.013 (±0.004)	0.500 (±0.300)
Zeon-1	0.021 (±0.006)	0.808 (±0.450)
	maize chromosome 10S (*orp2*)	
Ji-3	0.065 (±0.008)	2.500 (±0.600)
Milt-1	0.010 (±0.004)	0.385 (±0.300)
Opie	0.008 (±0.003)	0.308 (±0.225)
Prem-1	0.005 (±0.002)	0.192 (±0.150)
Ji-4	0.007 (±0.002)	0.269 (±0.150)
	0.004 (±0.000)	0.154 (±0.000)
Huck-2	0.051 (±0.000)	1.962 (±0.000)
Milt-2	0.082 (±0.010)	3.154 (±0.750)
	rice chromosome 8	
unclassified	0.007 (±0.003)	0.269 (±0.225)
Ty1-copia-like	0.007 (±0.003)	0.269 (±0.225)
OLDISREP	0.031 (±0.005)	1.192 (±0.375)
Dagul	0.099 (±0.011)	3.808 (±0.825)
Ty2-gypsy-like	0.002 (±0.002)	0.077 (±0.150)

Ty2-gypsy-like	0.002 (±0.002)	0.077 (±0.150)

***r1/b1* region**

LTRs	k (±SE)	Time (95% C.I.)
	maize chromosome 10L (*r1*)	
Opie-3	0.005 (±0.002)	0.188 (±0.150)
Ji-7	0.017 (±0.004)	0.662 (±0.278)
Ji-5	0.000 (±0.000)	0.000 (±0.000)
Ji-6	0.023 (±0.005)	0.892 (±0.338)
Opie-2	0.022 (±0.005)	0.831 (±0.368)
Huck-2	0.032 (±0.004)	1.215 (±0.323)
Ji-4	0.017 (±0.004)	0.650 (±0.278)
Ji-3	0.002 (±0.001)	0.088 (±0.098)
Tekay-like	0.023 (±0.003)	0.885 (±0.218)
Fasu	0.000 (±0.000)	0.000 (±0.000)
TIM	0.070 (±0.017)	2.688 (±1.305)
Opie-1	0.023 (±0.005)	0.885 (±0.338)
Ji-2	0.001 (±0.001)	0.023 (±0.045)
Fourf	0.009 (±0.003)	0.362 (±0.218)
	maize chromosome 2S (*b1*)	
Huck-3	0.014 (±0.003)	0.523 (±0.210)
Huck-5	0.008 (±0.002)	0.292 (±0.173)
Huck-4	0.011 (±0.003)	0.423 (±0.188)
Ji-9	0.019 (±0.004)	0.738 (±0.270)
Ji-10	0.009 (±0.003)	0.331 (±0.195)
Opie-4	0.053 (±0.007)	2.046 (±0.495)
Huck-7	0.008 (±0.002)	0.312 (±0.165)
Milt	0.043 (±0.008)	1.662 (±0.608)
	rice chromosome 4	
Huck	0.006 (±0.002)	0.231 (±0.150)
Tekay	0.021 (±0.002)	0.808 (±0.150)

***c1/pl1* region**

LTRs	k (±SE)	Time (95% C.I.)
	maize chromosome 9S (*c1*)	
Huck-1	0.028 (±0.005)	1.077 (±0.375)
Opie-1	0.023 (±0.004)	0.885 (±0.300)
Prem-1	0.000 (±0.000)	0.000 (±0.000)
Opie-2	0.023 (±0.004)	0.885 (±0.300)
Huck-2	0.013 (±0.003)	0.500 (±0.225)
Giepum	0.009 (±0.002)	0.346 (±0.150)
Opie-3	0.015 (±0.004)	0.577 (±0.300)
Huck 3	0.010 (±0.003)	0.385 (±0.225)
Cinful	0.009 (±0.002)	0.346 (±0.150)
Opie-4	0.001 (±0.001)	0.038 (±0.075)
Ji	0.000 (±0.000)	0.000 (±0.000)
	maize chromosome 6L (*pl1*)	
Ji-1	0.031 (±0.006)	1.192 (±0.450)
Huck	0.030 (±0.004)	1.154 (±0.300)
Dagaf	0.022 (±0.003)	0.846 (±0.225)
Ji-2	0.010 (±0.003)	0.385 (±0.225)
Ji-3	0.039 (±0.006)	1.500 (±0.450)
Ji-4	0.003 (±0.002)	0.115 (±0.150)
Ji-5	0.017 (±0.005)	0.654 (±0.375)
Fourf	0.017 (±0.004)	0.654 (±0.300)
Opie	0.003 (±0.002)	0.115 (±0.150)
	sorghum	
Kuheob	0.008 (±0.004)	0.308 (±0.300)
Deiho	0.017 (±0.004)	0.654 (±0.300)

_tb_ region

LTRs	k (±SE)	Time (95% C.I.)
	maize chromosome 1L (*tb1*)	
Eninu	0.004 (±0.002)	0.154 (±0.150)
Grande	0.002 (±0.002)	0.077 (±0.150)
Hopscotch	0.009 (±0.006)	0.346 (±0.450)
Huck	0.035 (±0.005)	1.346 (±0.375)
Ji	0.003 (±0.002)	0.115 (±0.150)
Milt	0.020 (±0.005)	0.769 (±0.375)
Yemi	0.000 (±0.000)	0.000 (±0.000)
Zeon-1	0.001 (±0.001)	0.038 (±0.075)
Zeon-2	0.077 (±0.012)	2.962 (±0.900)
	maize chromosome 5S (*tb2*)	
Bogu	0.002 (±0.002)	0.077 (±0.150)
Cinful	0.006 (±0.003)	0.231 (±0.225)
Ovamef	0.015 (±0.007)	0.577 (±0.525)
Prem-1	0.013 (±0.003)	0.500 (±0.225)
Zeon	0.005 (±0.003)	0.192 (±0.225)
	sorghum	
Grande	0.007 (±0.004)	0.269 (±0.300)

tbp region

LTRs	k (±SE)	Time (95% C.I.)
	maize chromosome 1L (*tbp1*)	
Huck-1	0.040 (±0.006)	1.538 (±0.450)
Huck-2	0.005 (±0.002)	0.192 (±0.150)
Huck-3	0.045 (±0.006)	1.731 (±0.450)
Ji-1	0.009 (±0.003)	0.346 (±0.225)
Ji-2	0.015 (±0.006)	0.577 (±0.450)
Opie	0.044 (±0.006)	1.692 (±0.450)
Rire	0.009 (±0.003)	0.346 (±0.225)
	maize chromosome 5S (*tbp2*)	
Huck	0.036 (±0.005)	1.385 (±0.375)
Opie-1	0.002 (±0.001)	0.077 (±0.075)
Ji-1	0.011 (±0.005)	0.423 (±0.375)
Opie-2	0.041 (±0.006)	1.577 (±0.450)
Ji-2	0.035 (±0.007)	1.346 (±0.525)
Giepum-1	0.024 (±0.006)	0.923 (±0.450)
Opie-3	0.004 (±0.002)	0.154 (±0.150)
	rice chromosome 3	
Ty3-*gypsy*-like	0.026 (±0.008)	1.000 (±0.600)

[a] The estimated distance of the LTR termini (*k*), based on Kimura's two-parameter model (K2P), and its standard deviation (SE) as described in Kumar et al. (2001).
[b] The estimated time of LTR retrotransposon insertion in millions of years ago and the 95% confidence interval (C.I.).
