# Supplementary material for: Retrotranspositions in orthologous regions of closely related grass species
Source: BMC Evol Biol. 2006 Aug 16;6:62. doi: 10.1186/1471-2148-6-62 (PMC1560396; doi:10.1186/1471-2148-6-62)
Supplement: Additional file 2 — Cladogram of auxin-related genes. The relationship of the auxin-related genes is presented as a cladogram resulting from maximum parsimony analysis using branch-and-bound search option. [file 1471-2148-6-62-S2.pdf]

Additional file 2 for "Retrotranspositions in orthologous regions of closely related grass species" by Du et al. BMC Evolutionary Biology #4916306119647011.

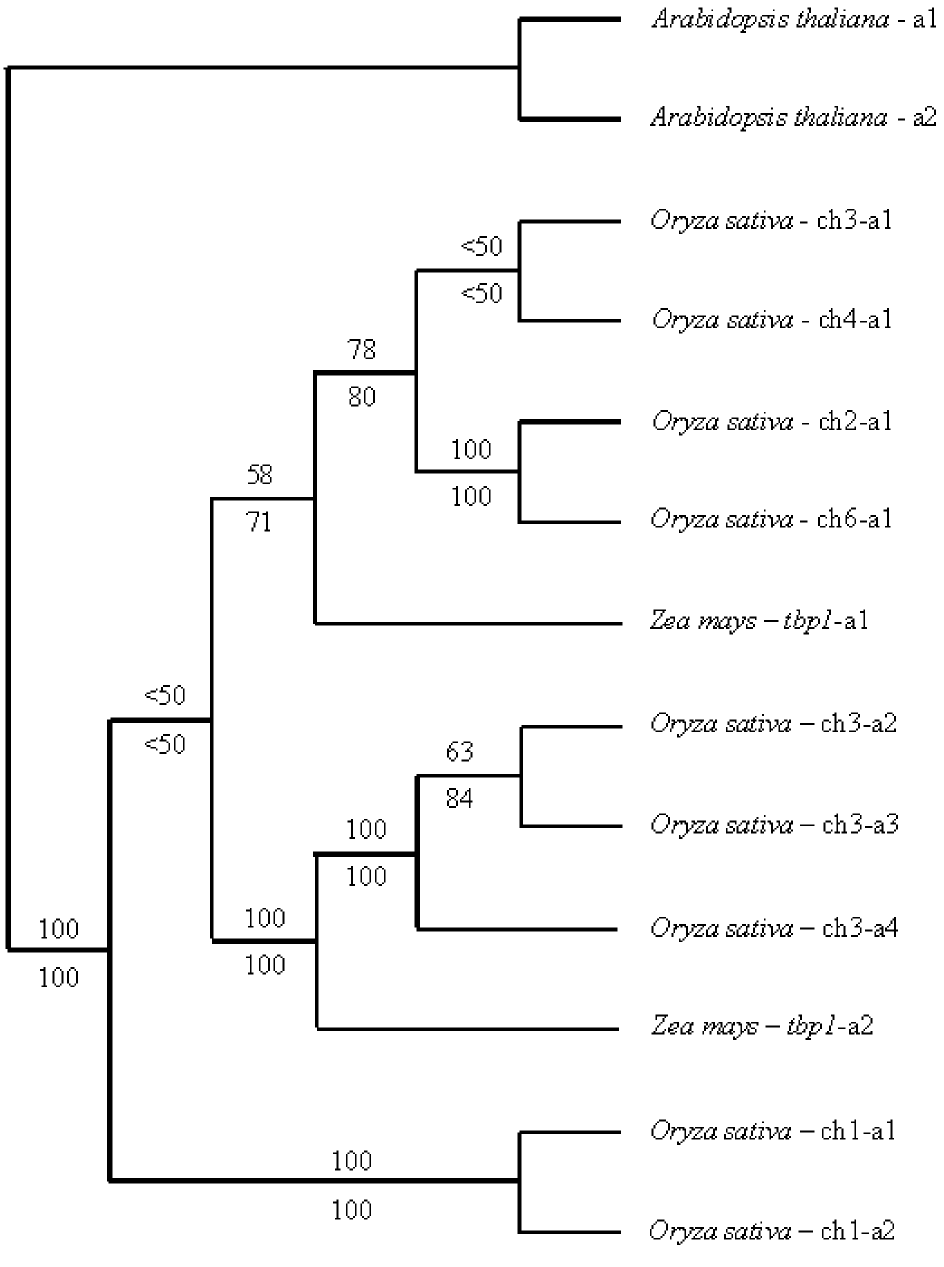

Cladogram of the auxin-related genes resulting from maximum parsimony analysis using branch-and-bound search option. Numbers at the internodes refer to bootstrap proportions (upper number based on data set with missing characters excluded, 220 total; lower number based on data including all characters, 1044 total; heuristic search performed with 1000 pseudoreplicates).
